# Giant Malignant Phyllode Tumor: A Case Report

**DOI:** 10.7759/cureus.33016

**Published:** 2022-12-27

**Authors:** Orjuana Khudari, Safaa S Allehyani, Moayad Alrefaie

**Affiliations:** 1 General Surgery, Al-Noor Specialist Hospital, Department of General Surgery, Makkah, SAU; 2 Breast Surgery, Al-Noor Specialist Hospital, Department of General Surgery, Makkah, SAU

**Keywords:** report, case, tumor, phyllode, malignant, giant

## Abstract

Phyllodes are an uncommon type of fibroepithelial neoplasm of the breast, which account for only 0.3 to 0.5% of all breast neoplasms. Management requires complete surgical excision with negative margins. Giant phyllode tumors portray a surgical challenge because complete surgical excision with negative margins is vital to reduce local recurrence and metastatic spread. Here, we report a case of giant malignant phyllode tumor, approached with wide local excision and negative margins were successfully achieved. The purpose behind this paper is to report the patient’s clinical history, presentation, intra-operative and histopathological findings, accompanied by a literature review to determine the significance of this finding and the approach in management.

## Introduction

Phyllodes tumors and fibroadenomas are fibroepithelial breast tumors, which are biphasic neoplasms distinguished by proliferation of both epithelial and stromal components. Phyllodes tumors are a rare fibroepithelial neoplasm comprising 0.3 to 0.5% of all breast tumors and around 2.5% of fibroepithelial tumors of the breast [1]. 

Originally, they were known as cystosarcoma phyllodes by Johannes Müller back in 1838 due to the tumor’s fleshy appearance and tendency to contain macroscopic cysts. Over time, the terminology has evolved, with the application of over 60 synonyms before the World Health Organization (WHO) settled with the term phyllodes tumors [2].

Moreover, the WHO sub-classified phyllodes tumors histologically in 2012 as benign (60-75%), borderline (15-20%), or malignant (10-20%), based on their histopathological features [2].

The mainstay of management of phyllodes tumors is complete surgical excision, either by mastectomy or wide local excision, to achieve histologically clear margins despite the surgical challenges, as it’s crucial to decrease local recurrence and metastatic spread [2].

Here, we report a case of malignant phyllode tumor that was treated with wide local excision, resulting in clear negative margins and good outcome with no recurrence.

## Case presentation

A 38-year-old female patient, not known to have any medical illnesses prior to presentation, presented to our breast and endocrine surgery clinic with a large ulcerating right breast mass. According to the patient, the mass was small when it was first noticed about 20 years back, growing in progression over the years, yet patient did not seek any medical advice previously.

Patient denied history of other masses or constitutional symptoms. Patient also denied family history of similar presentation, personal or family history of malignancy. Upon assessment, patient was average risk for breast cancer.

**Figure 1:**
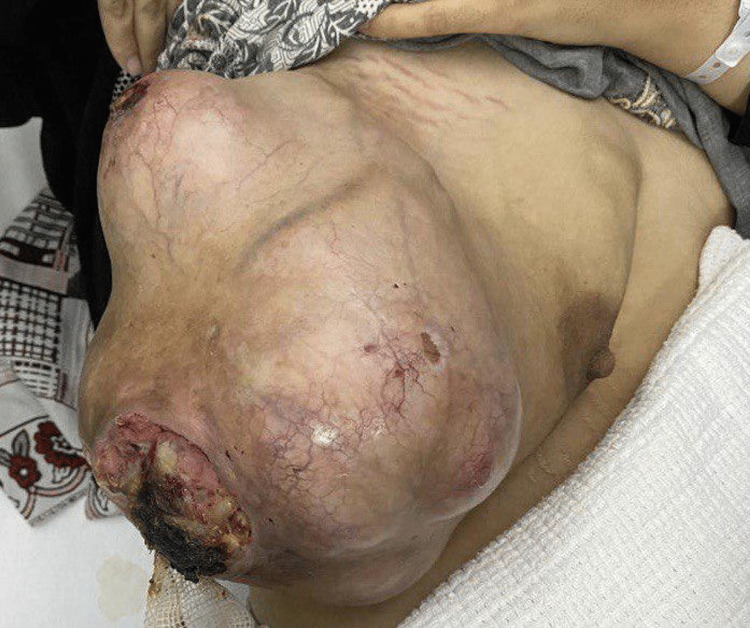
Pre-operative picture of patient's right breast mass.

On physical examination, the patient had a large right breast mass about 20 cm x 15 cm, found in the upper and lower outer quadrants of the breast, with two ulcers on the surface, pushing the nipple-areolar-complex medially with no retraction or nipple discharge. Palpable right axillary lymph nodes were also found on examination. However, the left breast and axilla were unremarkable. Figure 1 shows a pre-operative picture of the patient's right breast mass.

Patient was then investigated, her laboratory work-up was unremarkable and within normal ranges. Her breast US reported: "right breast is mostly occupied by a huge mass displaying heterogenous echos and cystic components*.*" A mammogram could not be performed due to the hugeness of the mass.

Tru-cut biopsy of the right breast mass was proceeded with, histopathology reported: "spindle cell with myxoid storma which can be stromal component of fibro-epithelial lesion such as fibroadenoma, phyllodes tumour or less likely myxoid myofibroblastoma."

Staging studies were negative for distant metastasis.

**Figure 2:**
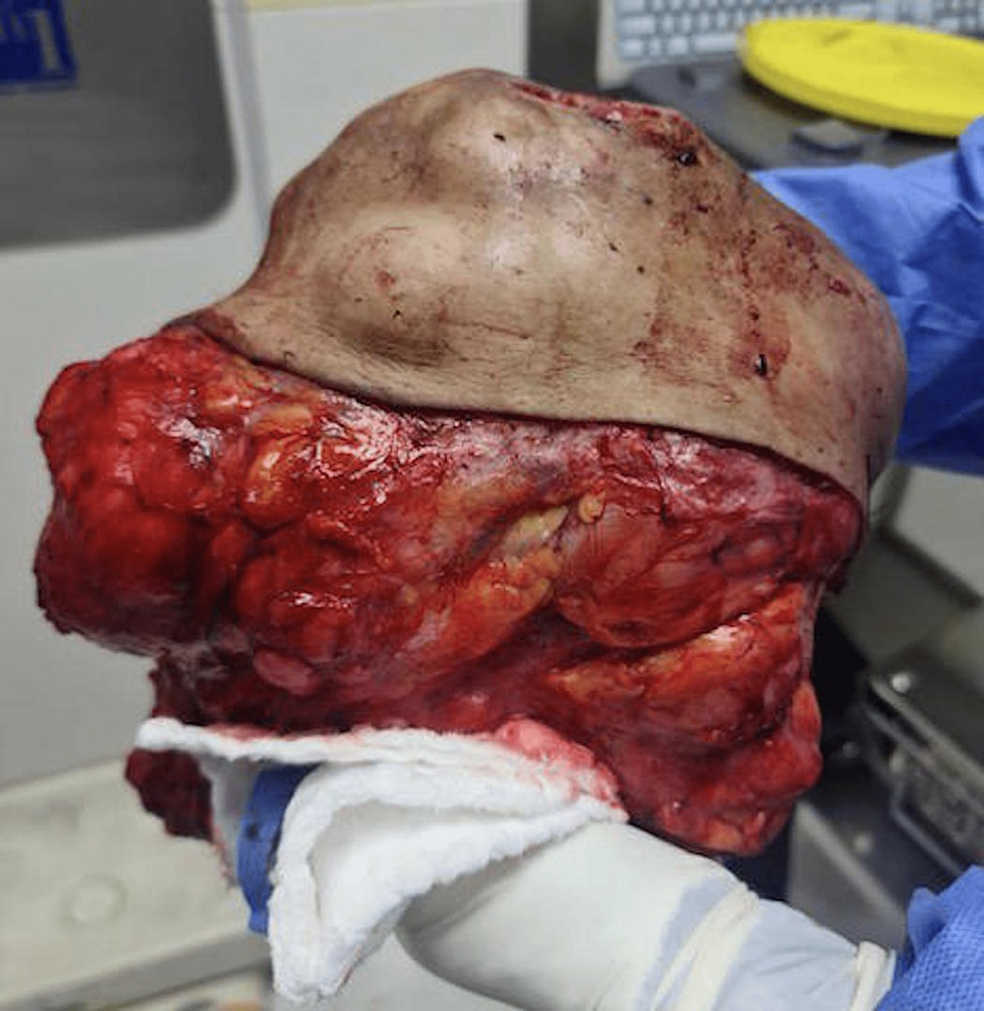
Intra-operative picture of specimen post wide local excision.

Patient was then operated and underwent right breast mass wide local excision with 1 cm margins of all borders removing the fascia and sparing the nipple-areolar-complex. Intra-operatively, the mass was found attached to the pectoral muscle which was included in the excision with its superficial layer to achieve margins. Primary closure of the defect was done with no need for skin grafts or flaps. Right axillary palpable lymph nodes excision was also carried out. Figure 2 shows an intra-operative picture of the specimen post wide local excision. Patient’s post-operative period was uneventful.

**Figure 3:**
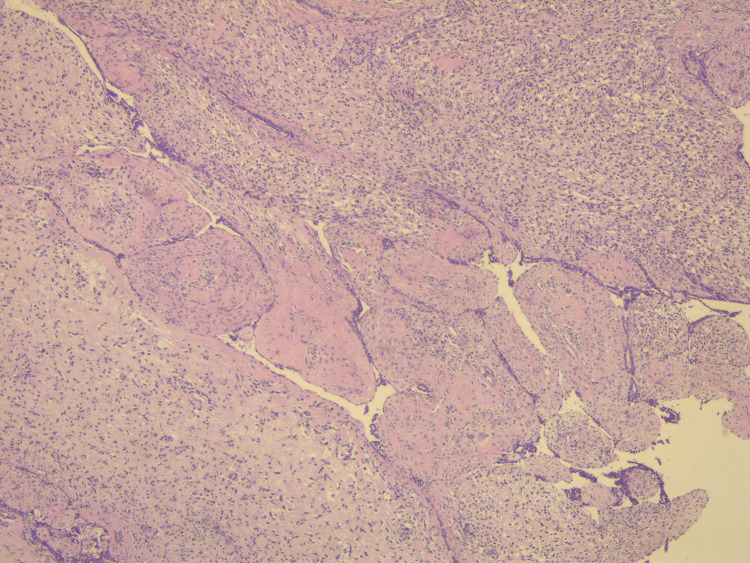
Histopathology of the excised breast mass; biphasic area.

**Figure 4:**
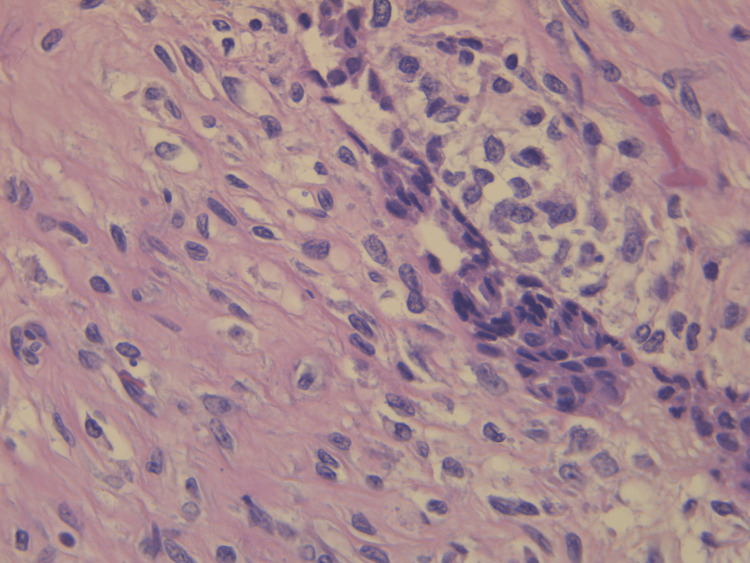
Histopathology of the excised breast mass; close-up.

**Figure 5:**
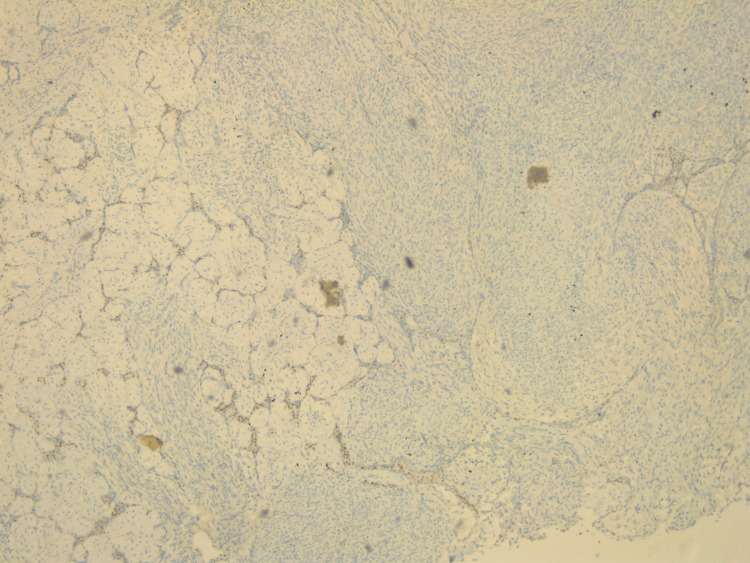
Histopathology of the excised breast mass.

Pathological evaluation of the specimen revealed a mass that weighed 3.8 kg and measured 20 x 17 x 15 cm. Grey-white circumstanced multi-nodular tumour in appearance. Histological examination revealed biphasic tumoural growth that was composed of stromal and epithelial components, with stromal overgrowth, cellularity and stromal atypia, with marked nuclear pleomorphism, hyperchromasia, irregular nuclear contours and focal area of sarcoma. Moreover, a high mitotic rate of 11/10 high-power field (HPF) was present. Figures 3, 4, 5 show the histopathology of the excised breast mass. 

The diagnosis based on histopathological examination was malignant phyllodes tumor with negative more than 1 cm margins. As for the axillary lymph nodes, histopathology revealed four reactive lymph nodes, negative for malignancy. The excised part of the pectoral muscle revealed fibrofatty and skeletal muscle tissue, also negative for malignancy.

Case was discussed on the tumor board and evaluated by medical oncologist; no further treatment was required of either chemotherapy or radiotherapy as negative margins were achieved with no need for further surgical intervention too, only follow-up with serial imaging was decided on. Upon six months follow-up, patient was symptom-free and imaging was unremarkable with no evidence of recurrence.

## Discussion

Phyllodes tumors are an uncommon type of fibroepithelial neoplasm of the breast, with an incidence of 1 per 100,000 women, and account for only 0.3 to 0.5% of all breast neoplasms [1]. As reported in the literature, they usually occur in the third or fourth decade of life. They are sub-classified by WHO into benign, borderline and malignant phyllodes tumors according to their histopathological features as their course is unpredictable. These five features include the degree of stromal cellular atypia, the mitotic activity per 10 HPF, the presence or absence of stromal overgrowth, infiltrative or circumscribed tumor margins and the nature of the tumor borders [2].

Phyllodes tumors can range in size but are frequently large, with a median of 4-5 cm, while sizes larger than 10 cm are considered giant phyllodes tumors. These tumors grow rapidly and the overlying skin may become shiny and translucent enough to reveal underlying veins. Eventually, giant phyllodes can grow and result in large, ulcerative, haemorrhagic lesions [3].

Even though imaging plays an elemental role in the diagnostic approach, confirmation of final diagnosis is achieved by histological examination and immunohistochemical analysis. Grossly, phyllodes tumors may be difficult to distinguish from fibroadenomas. They were described as round-to-oval multi-nodular masses with a grayish-white appearance bearing a resemblance to the head of a cauliflower. Phyllodes tumors grow radially, creating a pseudocapsule through which tongues of stroma may protrude and extend into adjacent breast tissue. Larger tumors may result in necrosis and hemorrhage. Microscopically, they harbor a characteristic leaf-like architecture consisting of elongated cleft-like spaces that contain papillary projections of epithelial-lined stroma with varying degrees of hyperplasia and atypia. Therefore, key components in differentiating phyllodes tumors from fibroadenomas are the stromal proliferation, cellular atypia and increased mitotic activity [3].

Complete surgical excision in management of phyllodes tumors is defined as the standard of care, regardless of its subclass. Most studies recommended a greater than 1 cm excision margins based on the evidence that local recurrence is higher in frequency with narrower surgical margins less than 1 cm ranging from 16.7% to 40%, while greater than 1 cm margins are more likely curative and decreases the risk of local recurrence [4].

According to the latest National Comprehensive Cancer Network (NCCN) of breast cancer, the management of phyllodes tumors larger than 3 cm is surgical excision with clean margins ≥1 cm without axillary staging whether the tumor is benign, borderline, or malignant. The guideline also suggests that when achievable, wide local excision is a better option over mastectomy since it preserves the overall architecture and integrity of the breast. However, since wide local excision requires free margins, it may not be possible or a challenge to perform a safe procedure with tumors larger than 10 cm and so mastectomy should be considered in giant phyllode tumors [5].

Lymph node involvement is rare with phyllodes tumors even with a malignant subclass, so routine axillary lymph node dissection is not necessary but may be recommended in patients with palpable lymphadenopathy. According to the literature, palpable axillary lymphadenopathy can be identified in up to 10-15% of patients but <1% have pathological positive nodes [6].

There is currently a lack of consensus regarding hormonal therapy, radiotherapy and systemic chemotherapy recommendation for malignant phyllodes tumors [4]. Yet, some recommendations were made to administer radiotherapy when adequate surgical margins cannot be achieved. Adjuvant radiotherapy and chemotherapy may be also used in patients with high-grade tumors with high mitotic rate, positive surgical margins, or postoperative recurrence, however their role is undefined [1].

Since phyllode tumors are locally recurrent tumors, a six-month interval follow-up is recommended for the first two years due to high chances of recurrence in that period [7]. Based on the literature, local recurrence was associated with inadequate surgical excisions, higher mitotic activity, stromal cellular atypia and necrosis rather than with tumor size. However, in multivariable analysis, surgical margins were found and established to be the only independent predictive factor for local recurrence [8].

In our reported case, with pre-operative planning and despite the large size of the tumor which measured 20 x 17 x 15 cm, wide local excision with negative more than 1 cm margins was successfully achieved, with no local recurrence on the first six-month follow-up.

## Conclusions

Phyllodes tumors are uncommon fibroepithelial breast tumors, which are sub-classified according to their histopathology. However, management of choice, despite histological sub-class, is complete surgical excision, either by mastectomy or wide local excision with more than 1 cm margins to decrease risk of local recurrence. To conclude, once a diagnosis of malignant phyllode tumor is made, pre-operative planning should favor wide local excision with negative margins whenever possible despite the surgical challenges, as they are curative if achieved and would maintain overall breast architecture even in giant phyllode tumors as the learning point and purpose of the report.
